# Dermoscopy of Pigmented Purpuric Lichenoid Dermatitis of Gougerot and Blum in an HIV-infected Patient

**DOI:** 10.5826/dpc.1004a75

**Published:** 2020-10-26

**Authors:** Pedro Miguel Garrido, Pablo Espinosa-Lara, Marta Aguado-Lobo, Luís Soares-Almeida, João Borges-Costa

**Affiliations:** 1 Dermatology Department, Centro Hospitalar Universitário Lisboa Norte, EPE (CHULN), Lisbon, Portugal; 2 Dermatology University Clinic, Faculty of Medicine, University of Lisbon, Portugal; 3 Dermatology Research Unit, Instituto de Medicina Molecular, University of Lisbon, Portugal; 4 Instituto de Higiene e Medicina Tropical (IHMT), Universidade Nova de Lisboa, Lisbon, Portugal

**Keywords:** dermoscopy, pigmented purpuric dermatosis, pigmented purpuric lichenoid dermatitis of Gougerot and Blum, human immunodeficiency virus

## Introduction

Pigmented purpuric lichenoid dermatitis of Gougerot and Blum is an uncommon variant of pigmented purpuric dermatosis (PPD) characterized by confluent purpuric papules and plaques on the legs [1]. The overlap of the clinical features of the subtypes of PPD usually hinders its diagnosis.

## Case Presentation

A 32-year-old black male, born in a West African country, reported the development of multiple asymptomatic brown-to-violaceous plaques on the legs in the previous 2 months. He was diagnosed with human immunodeficiency virus (HIV) type 1 infection 9 years before but had bad compliance with highly active antiretroviral treatment (HAART). One month before noticing the development of skin lesions, HAART was reinitiated with emtricitabine, tenofovir and dolutegravir. At the time of presentation, CD4+ lymphocyte count was 870 cells/mm^3^ and viral load was 22 copies/mL. Serologies for syphilis and hepatitis B and C virus were negative.

**Figure 1:**
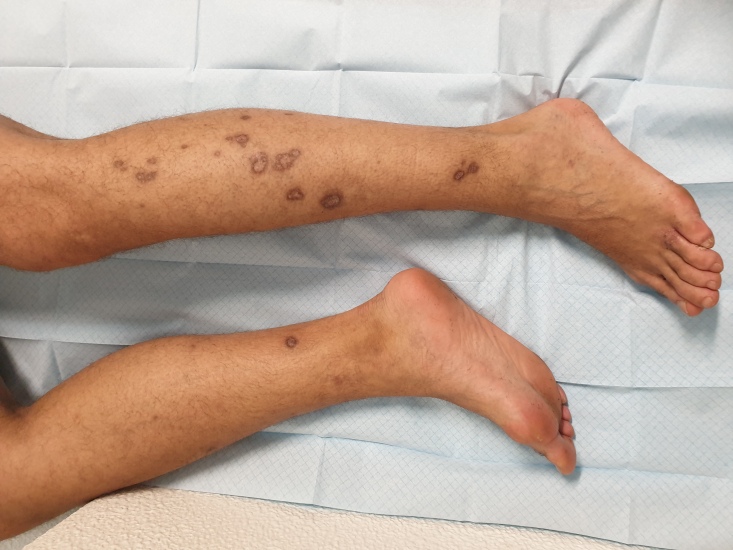
Clinical findings: red-brown nummular plaques with a lighter central area surrounded by a narrow erythematous-violaceous border on the legs.

On skin examination, the patient had multiple red-brown nummular plaques with an oval-to-irregular outline on the legs (Figure 1). The lesions measured 1 to 3 cm in diameter, had a lighter central area, and were surrounded by a narrow erythematous-violaceous border.

**Figure 2:**
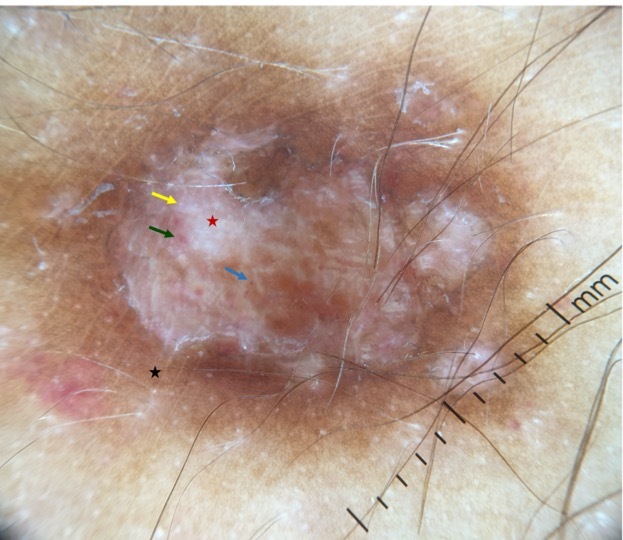
Dermoscopic findings: diffuse coppery-red structureless background (black star) permeated by a brownish-to-gray network with ill-defined borders and a polymorphic pearly white central area (red star) with polygonal-to-oval red and gray dots (blue and yellow star, respectively), red globules (green arrow) and patches.

Dermoscopy revealed a diffuse coppery-red structureless background permeated by a brownish-to-gray network with ill-defined borders and a polymorphic pearly white central area with polygonal-to-oval red and gray dots, globules, and patches (Figure 2).

**Figure 3:**
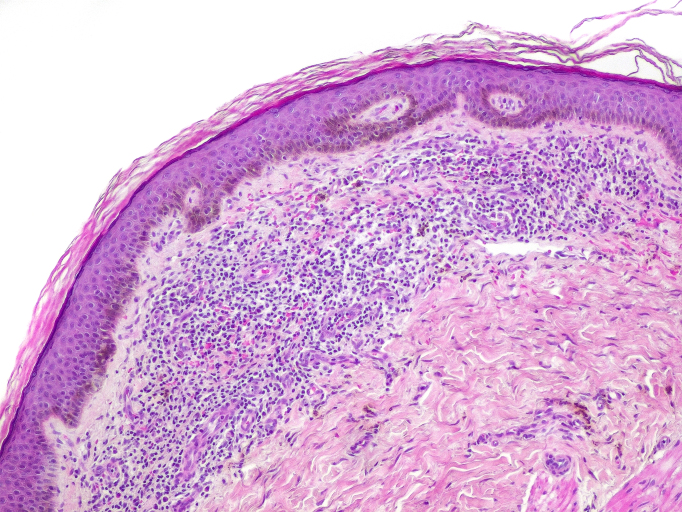

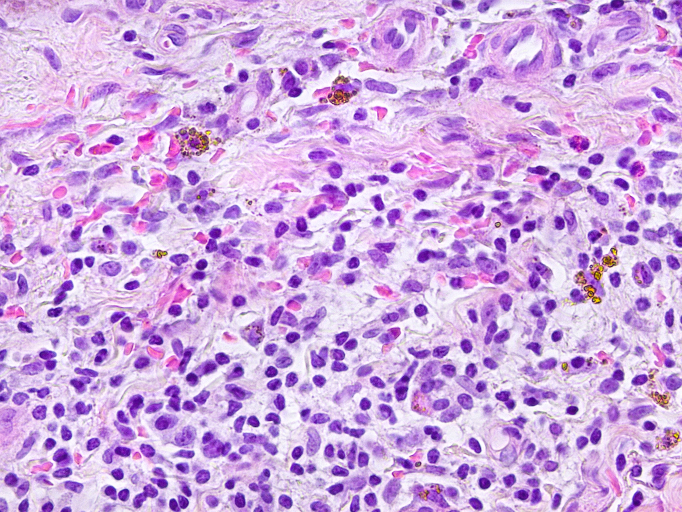

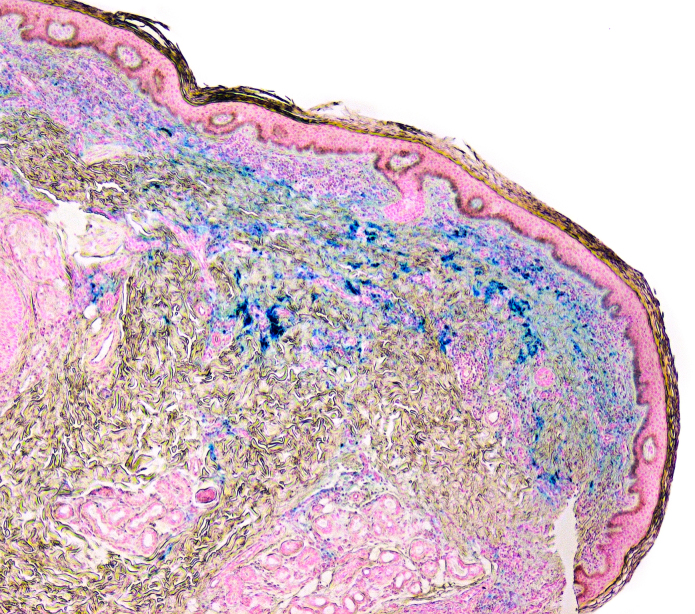
Histopathologic findings. (A) Dense band-like lymphocytic infiltrate in the upper dermis and increased number of dilated blood vessels. (B) Endothelial cell swelling and extravasation of red blood cells with hemosiderin inside of macrophages. (C) Hemosiderin deposition in the upper dermis. (A,B) H&E stain; original magnifications ×40, ×100. (C) Perls Prussian blue iron stain; original magnification ×40).

Histopathologic examination revealed a dense band-like lymphocytic infiltrate in the upper dermis, endothelial cell swelling and extravasation of red blood cells with hemosiderin inside of macrophages. There was no visible vessel wall damage (Figure 3).

## Conclusions

The clinical diagnosis of pigmented purpuric lichenoid dermatitis of Gougerot and Blum is challenging, as this dermatosis can closely resemble Kaposi sarcoma—an important differential diagnosis in HIV patients like ours—cutaneous vasculitis, and other PPDs, particularly lichen aureus. Dermoscopy is a noninvasive technique that may increase accuracy in the differential diagnosis. One previous report described its dermoscopic findings [2]. In comparison, our case stands out because of the coppery-red structureless background and the pearly white central area. The differences in the clinical presentation and dermoscopic findings correlate with the variability in histopathologic features: the presence of a coppery-red structureless background results from the lichenoid infiltrate and the extravasation of red blood cells with hemosiderin deposition in macrophages; the pearly white central area is explained by the increased number of hemosiderin-laden macrophages on the dermis.

PPD has been associated with many conditions, such as hypertension, diabetes mellitus, and autoimmune diseases [1]. Although the etiology of PPD remains unknown, previous reports suport a role for cell-mediated immunity on its pathogenesis [1]. In this case, skin lesions developed after initiation of HAART, alongside viral suppression and immunity improvement. Pigmented purpuric lichenoid dermatitis of Gougerot and Blum may therefore be a consequence of the immunity imbalance associated with the immune reconstitution inflammatory syndrome.
